# Advancing the Study of Glioblastoma Through 3D Tumor Models

**DOI:** 10.3390/cancers18040668

**Published:** 2026-02-18

**Authors:** Karen Salmeron-Moreno, Josephine Buclez, Chris Donghyun Kim, Karthik Papisetty, Thomas McCaffery, Fadi Jacob, Rommi Kashlan, Hithardhi Duggireddy, Karthik Valiveti, Justin Maldonado, Gustavo Pradilla, Tomas Garzon-Muvdi

**Affiliations:** Department of Neurological Surgery, Emory University, Atlanta, GA 30322, USA; ksalmer@emory.edu (K.S.-M.); jbuclez@emory.edu (J.B.); chris.kim3@emory.edu (C.D.K.); spapise@emory.edu (K.P.); tmccaf2@emory.edu (T.M.); fadi.paul.jacob@emory.edu (F.J.); rdkashl@emory.edu (R.K.); hduggir@emory.edu (H.D.); kvalive@emory.edu (K.V.); jmald1@lsuhsc.edu (J.M.); gpradil@emory.edu (G.P.)

**Keywords:** 3D systems, cancer, glioblastoma, spheroids, organoids, tumor microenvironment, bioprinting, tumor-on-a-chip

## Abstract

Glioblastoma (GBM), the most aggressive primary brain malignancy, remains a challenge to experimentally model. Accurately modeling the intra- and intertumoral heterogeneity of GBMs is essential for enhancing the predictive power of preclinical models and improving the effectiveness of current therapies. This review highlights recent advances in 3D tumor modeling, which accurately replicate the structural, cellular, and biochemical complexity of GBMs. We examine their translational potential and discuss current barriers to clinical translation.

## Simple Summary

Glioblastoma is an aggressive brain tumor characterized by marked cellular plasticity and molecular heterogeneity, which drive therapeutic resistance and near-universal recurrence. To better reflect this complexity, researchers have developed three-dimensional tumor models that recreate key features, such as cell interactions and the biochemical environment. This review aims to describe recent advances in these models, discuss practical considerations, and provide insight into the potential of three-dimensional tumor models to strengthen the connection between preclinical research and patient care.

## 1. Introduction

Glioblastoma (GBM) is the most aggressive primary brain malignancy in humans [1,2], defined by marked intratumoral heterogeneity, diffuse infiltration into surrounding brain parenchyma, robust treatment resistance, and near-universal recurrence [1,3,4,5,6,7]. Despite decades of molecular and clinical research, survival outcomes for patients with GBM remain poor, with a median overall survival of approximately 15 months, underscoring a persistent gap between molecular discoveries and therapeutic impact [8]. GBM is composed of multiple dynamic cellular states organized within a structured tumor microenvironment (TME) [9,10,11,12,13], an intrinsic complexity that remains a major barrier for accurate experimental modeling and a central challenge that must be overcome to bridge the translational gap [14,15].

Tumor behavior is guided by the TME, which encompasses components that suppress tumor immunosurveillance, promote angiogenesis, and induce pro-migratory signaling, among other pro-tumorigenic factors [16,17]. The highly complex architecture of the TME, characterized by abnormal vasculature, a heterogeneous mix of stromal, immune, and inflammatory cells, cancer stem cells, and a dense extracellular matrix (ECM) [18,19,20,21,22], combined with the need for adequate nutrient perfusion throughout both the tumor and surrounding tissue, illustrates how even minimal alterations in microenvironmental conditions can markedly influence tumor growth and invasive potential [23]. Accordingly, enhancing the biological accuracy of experimental models is essential to bolster their predictive validity and increase the likelihood of successful therapeutic translation.

Conventional two-dimensional (2D) cultures are capable of capturing certain angles of the microenvironmental gradients seen in vivo; however, the limited scalability and restricted applicability to patient-specific therapy response have driven the need for preclinical models that can reliably mimic the biological heterogeneity of each patient [24,25]. Three-dimensional (3D) tumor models enable controlled reconstruction of fundamental features of the TME, while integration of single-cell and multi-omics profiling has established quantitative maps to assess the fidelity with which these models replicate parental tumors, revealing translational strengths and biases [26,27,28].

In this review, we summarize current advances in 3D GBM models, with a focus on applications in translational research in the defiant landscape of brain tumor management.

## 2. Overview of Tumor Models

### 2.1. Two-Dimensional Tumor Models

2D cell cultures, developed as an adherent monolayer system, in which cells attach to a treated surface and grow side-by-side in a single-cell-thick layer that covers the available surface [24], have long served as fundamental tools in cancer research, providing an excellent foundation for mechanistic understanding of tumor cell signaling, yet they provide only partial insight into robust information on cell migration and invasion, cell–cell/cell–matrix interactions, and 3D tissue architecture [29,30,31,32,33].

The advantage of 2D models is that, under the right conditions, cell lines can proliferate in culture indefinitely; additionally, these models are cost-effective, commercially available, and do not pose ethical concerns related to animal welfare; however, the efficiency with which these models can be developed and maintained comes at the cost of reduced experimental reproducibility and risk of biological distortion [24]. Genetic drift can emerge in isolated cell lines due to the progressive accumulation of chromosomal alterations and point mutations over successive passages [34]. The use of differentiation-inducing DMEM media supplemented with 10% fetal bovine serum has been shown to alter transcriptional programs and epigenetic and functional states, often diminishing native or stem-like phenotypes and unpredictably modifying immune and invasive properties, further contributing to genomic instability [24,35]. As growth conditions vary between studies, the reproducibility of these experiments and the validity of their results should be interpreted with caution [24].

Patient-derived GBM cell lines, established from surgical specimens dissociated into single cells and subsequently propagated through clonal expansion in serum-free medium, may retain key oncogenic alterations of the parental tumor [36]. However, as intra- and intertumoral heterogeneity is a hallmark of GBM, the phenotype of these cultures is highly dependent on the specific cell isolated for propagation, leading to expected bias in the resulting culture [37]. Additionally, as these 2D cell cultures are composed mainly of tumor cells, they lack the other components of the TME and therefore fail to account for their functional contributions to tumor pathogenesis [24].

### 2.2. Three-Dimensional Tumor Models

**Figure 1:**
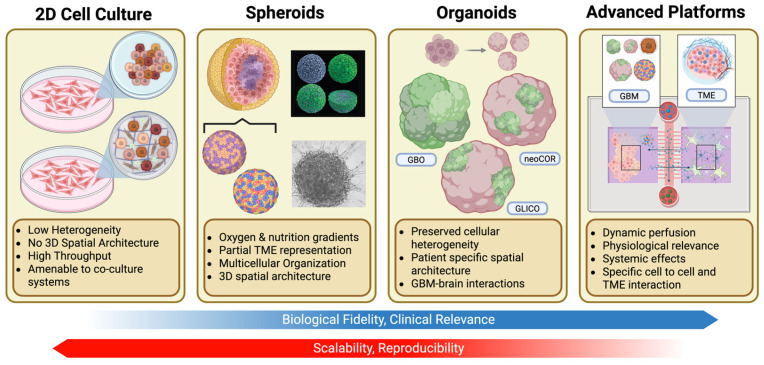
Overview of preclinical models for GBM research. Part of this figure was adapted from Allen Institute (2022) [46] and Witusik-Perkowska (2011) [47].

3D tumor models have the ability to replicate complex cellular interactions that are not captured in traditional 2D models [30,31,38,39,40,41,42,43,44,45]. Here, we describe four major classes of 3D GBM models—spheroids, organoids, bioprinter constructs, and microfluidic tumor-on-chip systems (Figure 1)—highlighting their technical characteristics and translational applications.

While complementary 3D approaches, such as tumor slice cultures and chorioallantoic membrane assays, are also available for GBM research, providing intermediate models between in vitro and in vivo studies by maintaining intact tumor tissue ex vivo or supporting rapid tumor engraftment and vascularization in vivo [48,49], they fall outside this review, which focuses on fully engineered and patient-derived in vitro platforms designed for controlled reconstruction of tumor architecture and microenvironmental dynamics.

#### 2.2.1. Spheroids

Spheroids are 3D cell aggregates generated by culturing tumor cells under non-adherent suspension conditions or within scaffold-based systems where they self-assemble into compact, spherical structures [30,44]. These models offer critical insight into the TME by replicating the coexistence of proliferating peripheral cells with quiescent or necrotic core populations, gradients of oxygen and nutrients, and complex cell–cell and cell–ECM interactions [31,44,50]. Moreover, by preserving the genetic and phenotypic diversity of the original tissue, patient-derived spheroids enable more accurate preclinical evaluation of therapeutic efficacy and drug responses [51,52].

Spheroids can be generated from cell lines, neural stem cells, or tumor tissue, offering considerable experimental flexibility, with marked variation in cellular composition and architectural organization depending on their derivation strategy [43,53]. They can be classified into four distinct groups: multicellular spheroids (MCSs), tumorspheres, tissue-derived tumorspheres (TDTSs), and organotypic multicellular spheroids (OMSs) [54].

MCSs originate from cancer cell lines without exogenous ECM (homotypic MCSs) or from co-cultures with immune, endothelial, or other stromal cells (heterotypic MCSs), forming in single-cell suspension cultures via forced or spontaneous aggregation under scaffold-free or scaffold-based conditions [54,55]. Tumorspheres are derived from primary tumors or cancer cell lines cultured under serum-free, low-attachment conditions, enriched to maintain undifferentiated states, appropriate for examining stem cell properties and for identifying therapeutic targets within this specific niche [56,57,58]. TDTSs emerge from mechanically or enzymatically dissociated primary tumor fragments cultured under serum-free conditions [56]. Although TDTSs preserve tumor-specific cell–cell interactions, they typically lack the heterogeneous stromal cell populations characteristic of GBM. As a result, these models facilitate a streamlined analysis of cancer cell-specific behaviors without the added complexity of the TME [59,60]. In contrast, OMSs are derived from unfragmented and undigested primary tumor samples, thereby preserving the native tissue microarchitecture, including vascular, immune, and stromal cell populations [61,62]. While OMSs are technically characterized as explant cultures rather than spheroids [56], they provide a superior physiological representation of the tumor but sacrifice consistency between samples [56,61]. A summary of spheroid models is presented in Table 1.

The increased physiological complexity of spheroids has exposed distinct patterns of drug (e.g., temozolomide) and radiation sensitivity [65,66], revealing resistance mechanisms masked in monolayer models, such as hypoxia, limited drug penetration, altered signaling, and the presence of dormant cell populations [66,67]. Additionally, GBM OMSs have been shown to preserve immunoreactivity for up to 16 weeks and the GFAP malignancy-associated marker over extended culture periods, in contrast to their rapid loss in 2D cultures [68]. Similarly, MCSs exhibit increased expression of P-selectin, a cell-adhesion molecule implicated in tumor invasion, underscoring the importance of spatial organization for TME signaling [69].

To boost biological relevance, co-culture spheroid systems incorporating astrocytes, microglia, and endothelial cells have also been developed to investigate the role of non-neoplastic cells in modulating GBM growth, immune evasion, and therapeutic resistance [70,71]. Human-induced pluripotent stem cell (hiPSC)-derived GBM spheroids have demonstrated intrinsic, tumor-specific migratory programs and facilitated the identification of actionable motility drivers, such as focal adhesion kinase (FAK) and CXCR4 signaling, whose pharmacological blockade suppresses spheroid migration [72]. Furthermore, scalable and reproducible GBM spheroids have enabled high-throughput assessment of therapeutic responses [73].

While spheroid models offer significant advantages, they also present notable limitations. Although endogenous ECM production partially replicates cell–ECM interactions, spheroids do not adequately reproduce the spatially patterned ECM architecture and exogenous matrix elements necessary to mimic the biochemical and biomechanical cues that regulate tumor growth, invasion, and drug response in vivo [32,74,75]. In addition, the absence of functional vasculature, the blood–brain barrier (BBB), angiogenesis, and immune infiltration limits their ability to accurately characterize tumor–host interactions [62,76]. In a therapeutic context, the 3D architecture of spheroids may also present challenges for uniform drug delivery and penetration [77]. Moreover, common assays, such as flow cytometry or multiplex screening used to characterize cell phenotypes, are also complicated by the need for spheroid dissociation [78].

Another important drawback is the variability in spheroid size, density, and reproducibility, which can affect experimental consistency [33,74]. These sources of variability are shared across multiple 3D tumor models, including organoids [79]. Scaffold-based systems have been employed to better replicate cell–ECM interactions, and standardized spheroid generation techniques have been encouraged to enhance reproducibility [33,44,45].

#### 2.2.2. Organoids

**Figure 2:**
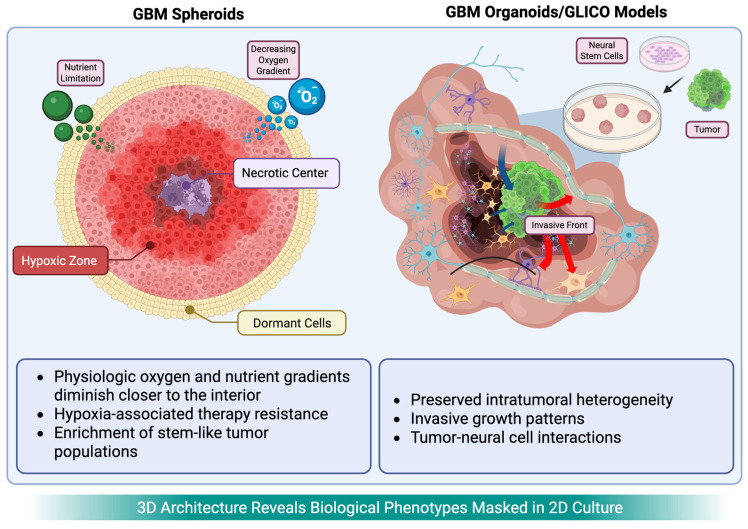
Comparative features of spheroid and organoid models.

Organoids are advanced self-organized cell-derived 3D models that recreate cellular diversity and spatial architecture in vitro by functioning as “mini-organs”, enabling more accurate simulation of tissue and tumor function [79,80]. Tumor organoids can be derived directly from both epithelial and non-epithelial tumor samples [79,81,82,83,84]. They differ from spheroids in their polyclonal composition and incorporation of surrounding stromal cells (Figure 2). These features make organoids particularly attractive for exploring intratumoral heterogeneity and patient-specific treatment responses [14,85,86].

Many organoid models have been developed specifically to study tumors of the central nervous system [87,88,89,90,91,92,93,94]. Organoids can usually be established from small diagnostic-type samples, on the order of a few millimeters up to 0.5 cm, provided that the tissue is appropriately handled [95]. For GBM, three principal organoid-generation strategies are outlined. First, patient-derived organoids (PDOs) are established from dissociated tumor cells embedded in ECM-rich cultures such as Matrigel—a solubilized basement membrane matrix—and maintained in defined media enriched with growth factors like WNT3A, EGF, NOGGIN, and RSPO1 [79]. While traditional PDOs contain predominantly malignant cells, to better emulate the TME, recent advances have enabled their reconstitution with stromal and immune components, such as tumor-infiltrating lymphocytes, macrophages, fibroblasts, and other tumor-associated cells, through either the expansion of endogenous immune cells or the incorporation of exogenous immune populations [84,85,96,97]. Individualized patient tumor organoids have been used to explore tumor heterogeneity, treatment response, and resistance mechanisms associated with temozolomide therapy [28,87,88,89,98,99]. However, PDO models that lack vascular networks remain limited in pharmacodynamic studies of therapies that rely on vascular permeability, lymphatic drainage, or systemic enzymatic activation [100,101]. Air–liquid interface and co-culture systems have provided intermediate solutions by supporting short-term maintenance of endogenous immune components [101].

Second, the cerebral organoid glioma (GLICO) platform combines patient-derived GBM cells or glioma stem-like cells with hiPSC-derived cerebral organoids to model tumor invasion within a neural context [93]. GLICO models preserve tumor heterogeneity and maintain interactions with astrocytes, neurons, and glial populations, recapitulating the 3D cytoarchitecture, cell–cell interactions, and microenvironment [88,91], making them particularly valuable for investigating tumor infiltration patterns [91,94]. However, GLICO models are constrained by batch-to-batch inter-organoid variability [85,102], absence of mature immune and vascular components [102], and extended culture timelines, often exceeding 60–90 days, which limits scalability for high-throughput screening [88,102].

Third, genetically engineered cerebral organoids generated via CRISPR/CAS9-mediated introduction of oncogenic drivers commonly observed in GBM, such as TP53 inactivation, PTEN loss, EGFRvIII amplification, or PDGF pathway activation, enable the spontaneous emergence of GBM hallmarks within an otherwise normal neural environment, providing controlled systems for studying gliomagenesis and early tumor evolution [74,93,103].

Despite these advances, several challenges remain. Growth factor-rich culture media used in traditional protocols represent a major limitation, as they drive clonal expansion of highly proliferative neoplastic cells and consequently reduce the representation of non-malignant cellular compartments [87]. Current efforts aim to enhance long-term viability through the incorporation of vascular networks, including co-culture with endothelial cells and engineered perfusion systems; additionally, upcoming research will focus on incorporating mature cell types to model later neurodevelopmental stages and enhance translational relevance [102]. Groundbreaking work utilizing pan-omics is continuously improving our understanding of tumor cell spatial interactions within the TME [104,105,106].

#### 2.2.3. Bioprinting

Over the past decade, 3D bioprinting models of GBM have emerged as powerful constructs for the study of tumor invasion, treatment response, and tumor–stroma interactions, with unprecedented architectural control, scalability, and biological relevance [107]. By enabling the layer-by-layer deposition of living cell-laden biomaterial (bioinks) into tissue-like constructs with defined geometry and spatial patterning [108], 3D bioprinting emulates key TME features [107,109].

A broad range of bioinks has been adopted for neural and GBM bioprinting applications. Natural polymers, such as hyaluronic acid (HA), silk, and Matrigel, provide ECM-relevant signaling, whereas synthetic materials, including methacrylate HA derivatives and polyethylene glycol, can be combined to balance printability, mechanical integrity, and bioactivity [107]. Multiple fabrication strategies, like inkjet, extrusion, photo-curing, and volumetric-based bioprinting [110], have been successfully applied to generate models of neural tissue [108,111,112,113], and have even demonstrated neuronal network formation and synaptic activity [114].

To overcome the limited mechanical stability of HA alone, supportive components, such as alginate, gelatin, or composite formulations, are incorporated to modulate stiffness, porosity, and degradation, while preserving HA-mediated signaling [110,115,116]. For example, digital light processing-based bioprinting using composite hydrogels combining gelatin methacrylate and glycidyl methacrylate-hyaluronic acid has enabled precise control of matrix mechanics while retaining relevant biochemical cues [117]. 

A defining advantage of bioprinting is the ability to impose intentional spatial organization, allowing distinct cellular compartments to be patterned with oxygen gradients, nutrients, and therapeutic exposure that better reflect the in vivo heterogeneity of GBM [111]. Tumor cells can be co-printed with astrocytes, neural precursor cells, endothelial populations, and immune components in defined locations, and the millimeter-scale thickness of the printed constructs enables multicellular crosstalk and context-specific functional dependencies [117].

Looking forward, the emergence of four-dimensional (4D) bioprinting, in which printed constructs evolve over time via stimulus-responsive biomaterials or remodeling matrices, offers a path to model GBM dynamics such as therapy-induced state changes, matrix remodeling, and time-dependent shifts in mechanical cues within the same construct [108,111]. Light-responsive ECM-mimetic hydrogels that undergo reversible stiffening upon exposure to blue light and relaxation in the dark illustrate the feasibility of this approach [118].

Continued innovation in bioink chemistry and printing strategies, alongside standardized readouts for perfusion, tumor invasion, and drug penetration, should further enhance the biomimicry and translational utility of bioprinted GBM constructs [111].

#### 2.2.4. Microfluidic Tumor-on-a-Chip Systems

**Figure 3:**
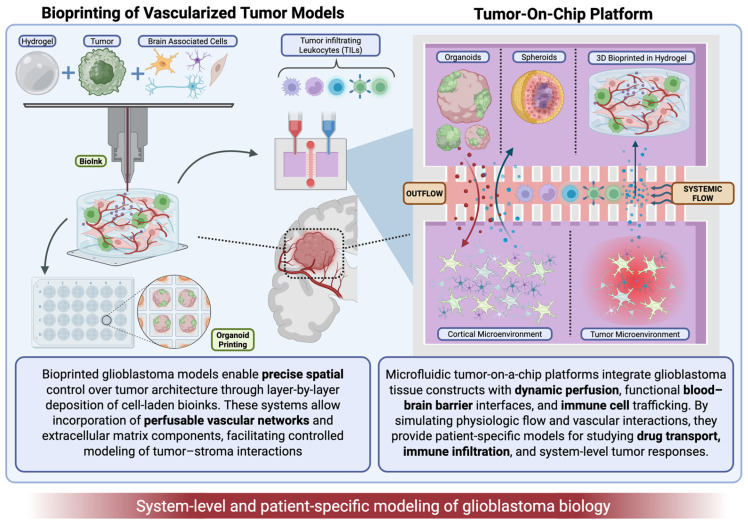
Emerging bioengineered models for translational tumor modeling.

Microfluidic tumor-on-a-chip models integrate microfluidic engineering with 3D tumor cultures, creating a dynamic in vitro model that enables controlled perfusion, spatial organization, and real-time modulation of biochemical and biomechanical cues. These function synergistically with 3D bioprinting by providing perfusable structures that support sustained nutrient delivery, drug exposure, and cellular trafficking (Figure 3) [18].

These systems are designed with microscale channels and chambers that enable precise control over interstitial flow, nutrient and oxygen gradients, and continuous drug perfusion, facilitating replication of the hypoxic and perivascular niches and diffusion-limited tumor regions that are difficult to achieve in static cultures [95,119].

Many microfluidic devices are fabricated in-house, offering the advantage of tailoring model parameters to suit specific experimental needs [23], including GBM invasion and interactions with the BBB [120,121,122]. These models have been used to induce pseudopalisading necrosis and migratory phenotypic transitions, allowing region-specific assessment of tumor viability [95,119,123]. Microfluidic models have also been applied to investigate angiogenic sprouting, vascular responses to anti-angiogenic therapies, and, more recently, to evaluate immunotherapeutic strategies in GBM. When integrated with 3D bioprinting, these systems have successfully reproduced patient-specific responses to chemotherapy and radiation [124,125].

The role of liquid biopsy has been established as a minimally invasive tool to study specific biomarkers released by tumors into the bloodstream, urine, and particularly cerebrospinal fluid for GBM [126,127,128]. However, low concentrations of tumor biomarkers have proven challenging to detect using traditional methods, limiting their sensitivity and specificity [125]. To address these limitations, microfluidics offers a potential solution through more effective isolation and detection of tumor-derived cells and biomarkers, thereby enhancing detection sensitivity and specificity [125]. Microfluidic platforms employ isolation strategies including affinity-based techniques that leverage tumor-specific surface markers, label-free physical separation methods based on size and deformability, and hybrid approaches to maximize biomarker recovery. Microfluidic devices have successfully integrated immunomagnetic selection with downstream molecular analyses to isolate extracellular vesicles and directly quantify drug resistance markers, such as MGMT mRNA, allowing for real-time monitoring of treatment response without invasive tissue sampling [129]. Furthermore, these platforms allow for multiplexed analysis of heterogeneous biomarker populations within a single sample, permitting simultaneous characterization of glioma stem cell signatures, mutational profiles, and therapeutic resistance mechanisms that may inform personalized treatment strategies [130]. Despite these advances, standardization of chip devices, integration of long-term vascular networks, and incorporation of mature immune and neural components remain active areas of development.

No single 3D model fully captures the complexity of GBM in vivo yet. Accordingly, selection of an appropriate model should be guided by the specific biological question and translational objective (Table 2). Equally important is the choice of culture media, which exerts a profound influence on cellular state, lineage fidelity, and microenvironmental interactions across all 3D models (Table 3).

## 3. Future Directions and Challenges

### 3.1. Current Landscape of 3D GBM Models

The modern landscape of 3D GBM modeling is no longer defined by the pursuit of a single optimal system but rather by the strategic alignment of multiple model architectures with specific biological and translational applications. Scalable spheroid and tumorsphere platforms remain widely used for probing hypoxia-driven resistance, diffusion-limited drug penetration, and collective invasion dynamics, where the controlled geometry enables reproducible phenotypic readouts at a throughput unattainable in vivo [28,77,131]. In parallel, patient-derived organoids and explant-based systems have emerged as high-fidelity platforms capable of preserving realistic intratumoral heterogeneity, transcriptional cell states, and treatment response patterns observed in parental tumors [87]. Brain organoid co-culture models, including GBM–cerebral–organoid assemblies, extend this by enabling direct interrogation of tumor infiltration within a human neural microenvironment that captures neuron–glia–tumor interactions inaccessible in traditional culture [87]. 3D bioprinted constructs and microfluidic tumor-on-a-chip systems further expand the design space by introducing spatial control over ECM composition, mechanical properties, and perfusion. This allows ex vivo replication of perivascular niches, drug gradients, and BBB interfaces [27,124]. The rapid expansion of this ecosystem has been accompanied by a shift toward adjacent assays using single-cell and spatial omics. These techniques are used to benchmark models against real tumors, identify ex vivo biases, and define the contexts in which each system replicates similar tumor biology [89]. These advances mark a transition from finding a single solution to GBM models ex vivo to using multiple frameworks depending on the experimental objectives.

### 3.2. Persistent Challenges

Despite rapid advances, several bottlenecks limit the recapitulation of key disease mechanisms. Parenchymal infiltration, typically extending centimeters along white matter tracts, remains compressed to millimeter-scale systems, limiting invasion modeling. The perivascular niche, where glioma stem cells interact with endothelial cells and pericytes under flow-dependent conditions, remains incompletely replicable without perfused vasculature [132]. BBB models drastically simplify transport physics; over 98% of therapeutic candidates fail BBB penetration, yet most platforms cannot predict this barrier accurately [133].

Immune modeling additionally poses challenges. GBM exhibits a “cold” TME characterized by myeloid-dominant immunosuppression, poor T-cell infiltration, and enrichment of regulatory T cells and myeloid-derived suppressor cells [134,135]. The tumor actively programs macrophages toward pro-tumor phenotypes through glucose-driven histone lactylation [136], while intrinsic low immunogenicity presents a barrier to immunotherapy [137]. Current models often lack immune components entirely or contain only transient populations that fail to establish stable immunosuppressive milieus.

Finally, GBM progression is driven by therapy-induced state transitions and clonal selection. Approximately 63% of patients experience expression-based subtype switching following treatment, with proneural-to-mesenchymal transitions representing a dominant axis of resistance and mortality [138,139]. Most models are optimized for short-term endpoints, making the tumor a static phenotype rather than a continuously evolving system.

### 3.3. Emerging Strategies

Current efforts focus on engineering systems that stabilize specific assays/experimental questions while minimizing variability. Perfused microfluidic BBB-GBM platforms featuring tumor spheroids with self-assembled vascular networks demonstrate physiological replicability for drug transport studies, with trafficking data correlating with in vivo intravital imaging [140]. Blood–tumor barrier organoids incorporating patient-derived GBM stem cells with brain endothelial cells, astrocytes, and pericytes demonstrate enhanced stemness and invasive behaviors matching in vivo observations [141].

Immune modeling has shifted toward preserving endogenous immune components in explants or incorporating mature microglia within organoid systems. PDOs retain non-neoplastic immune populations for at least two weeks [87], while tumor-myeloid organoids enable investigation of macrophage polarization effects [142]. Targeting the PERK enzyme, which regulates glucose metabolism and immunosuppressive activity in macrophages, has shown promise for overcoming immune evasion [136].

Single-cell and spatial transcriptomic benchmarking have become essential for validation. Engineered GBM organoids harboring subtype-specific mutations form xenograft tumors recapitulating the transcriptional and spatial landscape of human samples [143]. Automated image-based phenotyping and machine learning-driven segmentation have replaced subjective assays, while miniaturized formats enable high-throughput screening within clinically relevant timeframes [144].

### 3.4. Near-Term Outcomes

As these platforms grow, the near-term outcome is increasingly reliable model-to-patient alignment for specific therapeutic questions. Functional precision medicine pipelines combining comparative transcriptomics with organoid modeling have identified personalized targets resulting in radiographic disease stability [145]. Most notably, patient-derived GBM organoids have served as real-time avatars for chimeric antigen receptor (CAR) T cell therapy. Organoids treated with autologous CAR-T products showed target antigen reduction and cytolysis correlating with clinical engraftment in cerebrospinal fluid [146]. Single-cell benchmarking continues to define the context in which each model retains or loses intratumoral heterogeneity, enabling informed model selection based on the experiment’s requirements [147,148].

### 3.5. Long-Term Directions

The direction is now transitioning from short-term phenotypic assays toward systems modeling resistance as a dynamic, microenvironment-dependent process. Future models must capture therapy-induced mesenchymal reprogramming and GBM clonal evolution that drive treatment failure [139]. At scale, linking functional outputs to molecular profiling and clinical outcomes across shared datasets would enable these systems to define when and why standard treatments fail. Integration with circulating tumor DNA monitoring offers promise for tracking resistance emergence dynamically [149].

Ultimately, the long-term value of advanced GBM models lies in formalizing their resistance mechanisms into systems rather than approximating the tumor in full. Combined with computational integration and direct clinical correlation, these platforms hold the potential to transform GBM from an empirically treated disease into one amenable to precision-guided intervention.

## 4. Conclusions

3D tumor models have revolutionized GBM research by providing new methods for assessing tumor biology, drug resistance, and personalized therapeutic responses. These methods offer a complementary tool for studying the complexity of the TME, capturing aspects that are not easily recapitulated in traditional 2D models. Continued convergence of these technologies, together with increasing standardization of experimental parameters, is poised to advance GBM modeling toward next-generation precision oncology.

## Figures and Tables

**Table 1:** Classification of tumor spheroid models.

Spheroid Model	Cell Origin		Isolation Technique	Spheroid Composition		References
	Primary Cancer Cells	Cell Lines		Tumor Cells	Stromal Cells	
Multicellular Spheroids (MCSs)	+/−	+	-	++	+	[54,56,63,64]
Tumorspheres	+	+	Tumor tissue CSCs derived from enzymatic/ mechanical dissociation	++	-	[53,54]
Tissue-Derived Tumorspheres (TDTSs)	++	-	Excision with digestion & fragmentation	++	-	[53,54]
Organotypic Multicellular Spheroids (OMSs)	++	-	Excision without digestion	++	++	[54,56]

++ All models; + some models; +/− rare models; - no models.

**Table 2:** Comparison of key features, applications, and trade-offs across major 3D tumor models.

3D Tumor Model	Typical Cell Source(s)	Best Recapitulates	Use Cases/Readouts	Throughput	Time to Establish	Cost	Technical Complexity	Limitations	References
spheroids	Cell lines; patient tissue (TDTS, OMS); neurospheres	Hypoxia/nutrient gradients; rim-core organization; basic cell–cell/ECM interactions	Drug penetration and resistance screens; viability/proliferation; invasion; imaging; bulk/single-cell omics	High	Days–1 week	$	Low–Mod	No vasculature or BBB; ECM architecture limited; size variability	[30,32,33,42,43,44,47,50,53,55,56,59,60,61,63]
organoids	PDOs/IPTOs/ PDEs; iPSC/ASC-derived; direct tumor samples	Patient heterogeneity; 3D architecture; can include stromal/immune elements	Patient-specific drug response; histology; scRNA-seq/spatial; invasion (e.g., GLICO)	Medium	2–6 weeks	$$	Mod	Diffusion limits; Matrigel/batch variability; culture expertise	[62,64,79,80,82,84,85,86,87,88,90,91,93,94,98,99,102,104]
bioprinting	Defined patient-derived mixtures (tumor, endothelial, astrocytes, microglia, fibroblasts)	Spatial/architectural control; tunable ECM (e.g., HA-MA, gelatin, alginate); patterned heterogeneity	Vascular-like structures; gradient design; mechanics; migration/invasion under structure	Low–Medium	Days–2 weeks	$$$	High	Printer/bioink expertise; standardization; lower-scale throughput	[41,107,108,109,111,112,113,114,116]
tumor-on-a-chip	Spheroids/ organoids or dissociated cells in microchannels/gels	Perfusion and shear; controlled gradients; barrier models (e.g., BBB)	Real-time imaging; permeability/TEER; PK/PD; flow-based drug testing; transmigration	Low–Medium	Days–2 weeks	$$–$$$	High	Device fabrication; bubbles; lower throughput; specialized equipment	[18,19,23,40,122,124]

$ Low relative cost; $$ Moderate relative cost; $$$ High relative cost.

**Table 3:** Traditional composition for 3D tumor models.

Model	Structural Support	Media Components
Multicellular Spheroids/ Tumorspheres	None/Matrigel	Serum-containing or serum-free + growth factors
Tissue-Derived Tumorspheres/Organotypic Multicellular Spheroids	Endogenous ECM	Low-serum or serum-free
Patient-derived organoids	Matrigel/ECM-enriched hydrogels	Growth factors
Cerebral organoid assembloids	Endogenous ECM	Neural differentiation media + growth factors
Bioprinting	HA-based + composite bioinks	Model-dependent
Microfluidic tumor-on-chip	Composite hydrogels	Model-dependent

## Data Availability

No new data were created or analyzed in this study. Data sharing is not applicable to this article.

## Abbreviations

The following abbreviations are used in this manuscript:

2D	Two-dimensional
3D	Three-dimensional
4D	Four-dimensional
BBB	Blood–brain barrier
CAR	Chimeric antigen receptor
ECM	Extracellular matrix
GBM	Glioblastoma
GLICO	Cerebral organoid glioma
HA	Hyaluronic acid
hiPSC	Human induced pluripotent stem cell
MCS	Multicellular spheroids
OMS	organotypic multicellular spheroids
PDEs	Patient-derived explants
PDOs	Patient-derived organoids
TDTS	Tissue-derived tumorspheres
TME	Tumor microenvironment
